# Chronic Fructose Ingestion as a Major Health Concern: Is a Sedentary Lifestyle Making It Worse? A Review

**DOI:** 10.3390/nu9060549

**Published:** 2017-05-28

**Authors:** Amy J. Bidwell

**Affiliations:** Department of Health Promotion and Wellness, State University of New York at Oswego, 105G Park Hall, Oswego, NY 13027, USA; Amy.bidwell@oswego.edu; Tel.: +1-315-569-3543

**Keywords:** fructose, physical activity, metabolic syndrome, inflammation, insulin resistance, hyperlipidemia

## Abstract

Obesity contributes to metabolic abnormalities such as insulin resistance, dyslipidemia, hypertension, and glucose intolerance, all of which are risk factors associated with metabolic syndrome. The growing prevelance of metabolic syndrome seems to be an end result of our current lifestyle which promotes high caloric, high-fat foods and minimal physical activity, resulting in a state of positive energy balance. Increased adiposity and physical inactivity may represent the beginning of the appearance of these risk factors. Understanding the metabolic and cardiovascular disturbances associated with diet and exercise habits is a crucial step towards reducing the risk factors for metabolic syndrome. Although considerable research has been conducted linking chronic fructose ingestion to the increased prevalence of obesity and metabolic syndrome risk factors, these studies have mainly been performed on animals, and/or in a post-absorptive state. Further, the magnitude of the effect of fructose may depend on other aspects of the diet, including the total amount of carbohydrates and fats in the diet and the overall consumption of meals. Therefore, the overall aim of this review paper is to examine the effects of a diet high in fructose on postprandial lipidemia, inflammatory markers and glucose tolerance, all risk factors for diabetes and cardiovascular disease. Moreover, an objective is to investigate whether increased physical activity can alter such effects.

## 1. Introduction

The prevalence of obesity and obesity-related diseases in the United State and worldwide is increasing rapidly, with 67% of the population considered overweight and 33% obese [1]. Moderate obesity can contribute to chronic metabolic abnormalities characteristic of metabolic syndrome which include insulin resistance, dyslipidemia and hypertension [2]. Increased consumption of added sugar, specifically in the form of high-fructose corn syrup (HFCS) and sucrose, has paralleled the increased prevalence of metabolic abnormalities, and may be a contributing factor to the rise in the incidence of such disease-related risk factors [2].

The addition of fructose in the food supply became popular in the 1970s when fructose was used to produce high fructose corn syrup HFCS. HFCS can contain up to 90% fructose, however, most of the HFCS that is commercially sold contains 55% fructose and 45% glucose [2]. HFCS is frequently used as a sweetener in the food industry because it is cheaper to produce, has a long shelf-life, maintains long-lasting moisturization in industrial bakeries, and is sweeter than most other sugars [3].

There has been an increased interest in the potential role of these added sugars as a contributing factor to metabolic syndrome. When consumed in elevated concentrations, fructose can promote metabolic changes that may contribute to risk factors associated with metabolic syndrome as well as hyperuricemia, inflammation, and alterations in various metabolic hormones [4]. Today, the average American consumes ~94 g of added sugar per day. These values are significantly higher than the new dietary guidelines that state that no more than 25 g of added sugar should be consumed per day. The following review of literature will present evidence that fructose, either in the form of sucrose or HFCS, may cause substantial alterations in the risk factors associated with metabolic syndrome. Furthermore, partaking in an inactive lifestyle will also be addressed as increased physical inactivity may attenuate such risk factors.

## 2. Fructose Intake, Absorption and Metabolism

### 2.1. Fructose Intake

In recent years, manufacturers have been replacing HFCS with sucrose or other types of sugars, which is sometimes confusing the consumer, as one may think the product is somehow healthier than it really is. Sucrose, or table sugar, is a disaccharide composed of one glucose molecule and one fructose molecule, making it 50% fructose and 50% glucose [5]. Because sucrose has slightly lower concentrations of fructose compared to the 55/45 ratio of fructose to glucose in HFCS, often manufacturers will put more sucrose in the product in order to have it taste similar to products containing HFCS. Added sugar can be disguised as cane juice, evaporated cane juice, cane juice solids, cane juice crystals, or dehydrated cane juice, all made from sugar cane, therefore making them potentially as harmful as the now frowned upon HFCS.

### 2.2. Fructose Absorption

Fructose enters the brush border of the stomach in the form of either pure fructose, HFCS or as sucrose [6]. When fructose is ingested as a disaccharide in the form of sucrose, the sucrose must first be cleaved, via sucrose, into one molecule of glucose and one molecule of fructose before being metabolized. Fructose is then absorbed and transported through the enterocytes to the portal bloodstream by a fructose-specific hexose transporter, glucose transporter 5 (GLUT 5). Unlike glucose, which uses a sodium- and protein-based transporter molecule to assist the glucose with transport out of the enterocytes, the activation of GLUT 5 transporters is sodium–independent and does not require ATP hydrolysis [6]. Once inside the enterocytes, fructose diffuses across the basolateral pole of the enterocytes and into the portal circulation via glucose transporter 2 (GLUT 2) transporters [7].

Unlike glucose, fructose is incompletely absorbed in the enterocytes. The absorption capacity of fructose is limited to approximately 5–50 g at one time before some individuals suffer from symptoms of diarrhea and flatulence [8]. Ushijima et al. [9] showed that 80% of healthy adults experienced incomplete absorption when given 50 g of fructose, yet when fructose is consumed with glucose, the rate of absorption is increased [10]. Thus, when fructose is consumed as sucrose or as HFCS, more fructose is absorbed through the enterocytes. The improved absorption of fructose in conjunction with glucose may be due to the up-regulation of GLUT 5 receptors which is stimulated by elevated glucose absorption [8]. Once within the enterocytes, fructose can be easily converted into triglycerides (TGs). Specifically, intestinal TGs, in the form of chylomicrons, have been apparent in hamsters fed a high-fructose diet in as little as three weeks [11]. Moreover, chronic fructose feeding seems to be associated with increases in intracellular apoprotein-B48 (apoB-48) and enhanced intestinal enterocyte de novo lipogenesis (DNL) [11]. The intestinal overproduction of apoB-48-containing lipoproteins may be an important contributor to the elevation of circulating TG-rich lipoproteins, which may potentially lead to atherosclerosis.

### 2.3. Fructose Metabolism

Although fructose can be lipogenic within the enterocytes, fructose is also readily absorbed and stimulates lipogenesis within the hepatocytes [6]. Once fructose travels through the enterocytes and into the portal vein, it is readily absorbed by the liver via GLUT 2 transporters. Due to the high concentration of GLUT 2 transporters and fructokinase, there is a high affinity for fructose uptake in the liver [6]. Within the liver, fructose is rapidly converted to fructose-1-phospate via fructokinase. Fructokinase has a low affinity for fructose, resulting in rapid metabolism of fructose by the liver cells. Fructose is further metabolized into triose phosphates, glyceraldehyde and dihydroxyacetone phosphate [12]. The triose phosphate that is produced can then be converted to pyruvate and oxidized into carbon dioxide and water in the citric acid cycle or a portion of the triose phosphate can be converted to lactate and released into the systemic circulation [6]. A portion of the carbon derived from the triose phosphates can also enter the gluconeogenic pathway where it can be stored as glycogen to be later released as glucose [12]. This gluconeogenic process results in a small but measurable increase in systemic glucose concentrations [6].

**Figure 1:**
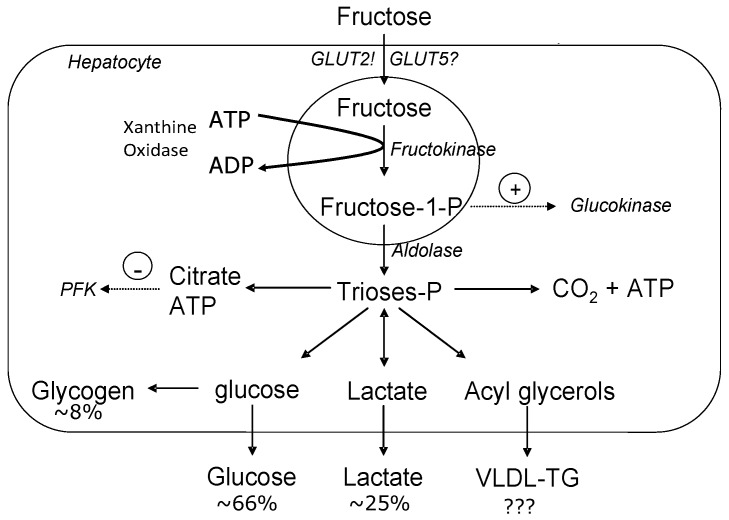
Metabolic fate of hepatic fructose. Fructose provides a high concentration of unregulated acetyl-CoA which can be converted to very-low density lipoprotein-triglyceride (VLDL-TG), glucose and/or lactate. (Figure adapted from Le and Tappy, 2006 [13]). GLUT: glucose transporter; PFK: phosphofructokinase.

Within the liver, fructose metabolism differs substantially from glucose metabolism in that entry of glucose into the glycolytic pathway is under the control of glucokinase which has a low affinity for glucose within the hepatocytes, and is dependent on the concentration of glucose [6]. Therefore, the rate of glucose phosphorylation varies with changes in glucose concentrations. Moreover, downstream, when fructose-6-phosphate is converted to fructose 1,6-bisphosphate, this reaction is catalyzed by phosphofructokinase (PFK), an enzyme regulated by the energy status of the cell. In particular, PFK is inhibited by elevations in ATP and citrate. This inhibition allows for a close regulation of glycolysis based on the energy status of the cell [12]. On the contrary, fructose is phosphorylated to fructose-1-phoshpate by fructokinase, but this rate-limiting enzyme does not have the tight regulation as seen with PFK [3]. Figure 1 depicts the metabolic fate of fructose within the hepatocytes. When acetyl-CoA combines with oxaloacetate to form citrate in the mitochondria, the carbon atoms can be used for DNL and then form long-chained fatty acids that are eventually esterified into TGs [12]. This large source of unregulated TG formation is unlike that of glucose metabolism which has a rate-limiting step to regulate it, preventing such effects.

## 3. Fructose-Induced Lipogenesis

The most detrimental aspect of fructose is its ability to be converted to fatty acids within the hepatocytes via DNL, as pictured in Figure 1. In rodents, a high-fructose diet (60% fructose) has been shown to increase intra-hepatocellular lipids as well as stimulate hepatic DNL within a few days [14]. When such diets are sustained over a prolonged period of time, high fructose or sucrose diets will induce hepatic stenosis and whole-body insulin resistance with a concomitant accumulation of intramyocellular lipids [15].

To date there is an abundance of research indicating that acute and/or chronic ingestion of fructose causes hyperlipidemia in rats [14,16,17] and in humans [18,19,20,21]. Faeh et al. [21] discovered that after six days of fructose loading, subjects’ plasma triglyceride concentrations were increased by 79% from baseline values, possibly due to the six-fold increase in fractional DNL [21]. It should be noted that the fructose load that was given (~210 g/day) in this study was an extremely high load and therefore may not be clinically relevant. Using a more clinically relevant fructose load, Swanson et al. [22] discovered that serum total and low-density lipoprotein (LDL) cholesterol levels were 9% and 11% higher, respectively, when consuming a high fructose diet compared to an isocaloric starch diet [22]. Furthermore, within the first 24 h, serum triglyceride levels in the fructose-fed group were significantly higher than the starch group, indicating that fructose induced hyperlipidemia can occur in as little as 24 h after the first fructose load [22]. In a slightly longer intervention, Bantle et al. [23] compared similar effects and found that a diet consisting of either 17% of energy from fructose or 17% glucose for six weeks was associated with elevations in fasting and postprandial TG concentrations [23]. These results were similar to those obtained by Schwarz et al. [24] in which elevated liver DNL was apparent in eight healthy men consuming 25% fructose/25% glucose mixture compared to a 50% complex carbohydrate mixture. It is important to mention that the above-mentioned study was one of the first studies to indicate an increase in DNL during a weight-neutral period, therefore demonstrating that the changes in DNL are not the result of increased weight but are from increased fructose consumption [24].

Although the research regarding fructose ingestion and fasting and postprandial lipogenesis is apparent in normal weight individuals, research is more limited in the obese population. Swarbrick et al. [18] investigated the metabolic effects of a high-fructose diet in seven overweight, post-menopausal women who consumed standardized, energy-balanced meals for 14 weeks. After two weeks on the diet, triglyceride area under the curve (AUC) was unchanged, however after week ten, triglyceride AUC values were 141% higher than at baseline. Additionally, fasting apolipoprotein B concentrations were increased by 19% compared to baseline. The authors speculated that the increases in fasting and postprandial TG concentrations were most likely due to stimulation of TG synthesis [18]. Likewise, Stanhope et al. [19] also investigated the overweight/obese population. After studying 18 post-menopausal women for 12 weeks, the high fructose group had elevated fasting and 24-h postprandial TG concentrations compared to the isocaloric glucose group [19]. Post-intervention, fasting apolipoprotein B, low-density lipoproteins (LDLs), small-dense LDLs, and oxidized LDLs were significantly higher in the fructose group compared to the glucose group. This study reitorates the fact that long-term consumption of fructose of ≥2 weeks negatively alters lipid remodeling in obese, post-menopausal women [19]. It seems that the mechanism by which fructose-induced lipemia occurs is a result of the carbon atoms from fructose being converted to fatty acids, skipping the rate-limiting step in glycolysis. Fructose increases DNL by increasing hepatic TG formation, however, fructose-induced hepatic DNL may also limit fatty acid oxidation as well. Fructose increases acetyl-CoA concentrations in the liver, subsequently leading to increased production of malonyl CoA, which inhibits the entry of fatty acids into the mitochondria [3]. Taken together, fructose indirectly inhibits fatty acid oxidation by increasing production of malonyl CoA, which decreases fatty acid transport into the mitochondria [3]. Malonyl CoA is an important intermediate to fructose-induced lipogenesis because acetyl CoA is added to long-chained fatty acids via malonyl CoA, therefore allowing fructose to provide carbon atoms for both glycerol, and the acyl portion of the acylglycerol molecule [3].

To better understand the hypothesis that fructose ingestion may also inhibit fat oxidation, Abdel-Sayed et al. [25] investigated whether a high-fructose diet (234 g) impaired lipid metabolism. After seven days on the high fructose diet, basal non-esterified fatty acid (NEFA) concentrations significantly decreased by 19.5%, net lipid oxidation by 21.3% and plasma β-hydroxybutyrate concentrations by 78.2%. After a period of lipid loading, the increase in net lipid oxidation and exogenous lipid oxidation were comparable between the two groups. However, after the mental stress, there was a markedly blunted stimulation of plasma NEFA and β-hydroxybutyrate release in the fructose group. The lower basal plasma NEFA concentrations indicated that an inhibition of adipose tissue lipolysis occurred after the high fructose diet. This phenomenon suggests that the decreased NEFA seen with the high-fructose diet was likely related to fructose-induced stimulation of hepatic DNL lipogenesis, and not secondary to an increased hepatic re-esterification. Additionally, the inhibition of lipolysis may, in turn, be directly responsible for lower whole-body net lipid oxidation following fructose loading since NEFA concentrations are the main determinant in this process [25].

## 4. Fructose and Postprandial Lipemia

Although research regarding fasting hyperlipidemia and fructose consumption has been well established, high postprandial triglyceride levels have been associated with the risk of coronary artery disease [26]. Hence, there is growing evidence linking increased postprandial TG concentrations with a pro-atherogenic state. This link may be due to lipoprotein remodeling induced by increased levels of very-low density lipoproteins (VLDLs) and mediated by cholesteryl ester transfer protein (CETP) and hepatic lipase. Both increased VLDLs and CEPT resulted in increased concentrations of small-dense lipoproteins and remnant-like lipoproteins [26].

When in the blood, TGs can be referred to as “triglyceride-rich lipoproteins” (TRLs) and consist of two main components: very-low density lipoproteins (VLDL) and chylomicrons. Very low-density lipoproteins are a result of hepatic synthesis and chylomicrons are produced by the gut postprandially in order to transport dietary lipids from the intestines to other locations in the body [26]. Therefore, TRLs can be produced exogenously from the diet or endogenously from the liver. Chylomicrons and VLDLs can then form intermediate-density lipoproteins catalyzed by lipoprotein lipase (LPL), an enzyme released from the capillary beds of adipose tissue and skeletal muscle [26]. Lipoprotein lipase, situated in the capillary endothelial, is responsible for hydrolyzing the TG into NEFA [27]. This pathway is up-regulated by insulin, which increases rapidly in response to a carbohydrate meal. In a fructose-rich diet, due to the suppression of insulin, reduced insulin concentrations may contribute to lower postprandial LPL activity. Research has indicated that glucose has a significantly greater postprandial LPL response than fructose [19], signifying that reduced TG clearance with chronic fructose ingestion might also contribute to the fructose-induced postprandial hypertriglyceridemia that is often evident in fructose-fed individuals [19]. Other studies have shown similar results in that fructose, not glucose, leads to an attenuated LPL response which potentiated postprandial lipidemia as the TG-VLDL and TG-rich chylomicron levels were significantly higher than in the glucose group [28]. The lower insulin concentrations seen with the fructose load led to a decreased production of LPL, resulting in impaired triacylglycerol clearance [28].

Often, consumption of fructose occurs in a postprandial state as the average Western diet is consumed every 3–6 h. Previous research has shown that a bolus of fructose in the morning with a subsequent meal at lunch stimulates lipogenesis and seems to be dose-dependent [20]. This same study found TG incremental AUC to be higher after a fructose bolus than a glucose bolus, signifying that fructose acutely and significantly increases lipogenesis in the morning and meals thereafter. The fructose-induced increase in lipogenesis displaced the use of stored TG for VLDL synthesis and the stimulation of lipogenesis represents an intracellular signal for the liver to esterify fatty acids from any source into TGs [20]. Similar results can be found in overweight and obese individuals [19]. Based on these findings, DNL and decreased lipoprotein lipase-mediated clearance may be a contributing factor to fructose-induced postprandial hypertriglyceridemia [19,20]. Although more research needs to be conducted in the postprandial state, when fructose is consumed in the morning, the succeeding meal will augment the postprandial lipidemia induced during the prior meal [20]. Possible mechanisms involved in the stimulation of fructose-induced postprandial lipidemia are: (1) the liver being the main site of fructose metabolism; (2) fructose bypassing the main rate limiting step of glycolysis, thus providing unregulated amounts of lipogenic substrates such as acetyl-CoA and glycerol-3-phosphate; and (3) fructose enhancing DNL when subsequent meals were ingested [19,20].

## 5. Fructose and Insulin Resistance

Type 2 diabetes is a progressive disorder that begins with the development of insulin resistance and potentially ends with pancreatic β-cell failure [29]. A dietary recommendation often proposed for patients suffering from type 2 diabetes is to ingest foods that do not cause an acute rise in insulin levels, therefore preventing over-stimulation of insulin secretion from the pancreas. Initially, fructose was a popular macronutrient choice for individuals with type 2 diabetes because fructose does not cause an acute rise in insulin due to the low glycemic index related to fructose. Although there is a blunted insulin response, fructose consumption has been associated with increased hepatic VLDL triglyceride secretion, and possibly decreased extra-hepatic clearance of very-low density lipoprotein-triglyceride (VLDL-TG), both of which are associated with the development of hepatic and adipose tissue insulin resistance. The VLDL-TG formed from fructose-induced hepatic DNL can be released into the systemic circulation, consequently leading to an increase in the levels of fatty acids in the circulation. Signaling abnormalities in adipocytes can also trigger lipolysis of TG stores and efflux of NEFA into the bloodstream, augmenting the problem [30]. NEFA in the bloodstream as a result of increased fructose-induced lipidemia may be a key mechanistic link between fructose consumption and insulin resistance, type 2 diabetes and metabolic dyslipidemia. These conditions are a result of increased ectopic storage of NEFA by non-adipose tissues such as liver and skeletal muscle where they are stored as TG or diacylglycerol. The exposure of these organs to increased concentrations of NEFA from fructose ingestion may reduce insulin sensitivity by increasing the intramyocellular lipid content [31]. Once stored as ectopic lipids, the fatty acids can interfere with the metabolic pathways of that tissue, resulting in fructose-induced insulin resistance [30].

In a healthy adult, insulin suppresses hepatic gluconeogenesis and glycogenolysis, however, in the insulin-resistant state, this suppression no longer occurs, causing a subsequent increase in glucose output from the liver [29]. Insulin resistance in fat cells reduces the normal effects of insulin on lipids and results in reduced uptake of circulating lipids and increased hydrolysis of stored TG. Increased mobilization of stored lipids in these cells elevates free fatty acids in the blood plasma, leading to reduced muscle glucose uptake and increased liver glucose production, all of which contribute to elevated blood glucose levels [32]. This chronic state of excess fatty acid release into the circulation can induce lipotoxicity, or pancreatic β-cell death. To compensate for the increased peripheral insulin resistance, the pancreatic β-cells increase in mass and secrete more insulin, resulting in hyperinsulinemia. Since the β-cells cannot compensate for the resistant state, hyperglycemia occurs. Hyperglycemia further damages the β-cells, resulting in glycotoxicity, leading to a progressive loss of the pancreatic islet β-cells manifesting into type 2 diabetes [29].

The molecular mechanisms underlying fructose-induced insulin resistance are not completely understood but may be similar to that of a high-fat diet. Both high-fructose and high-fat diets interfere with insulin signaling at common steps in skeletal muscle [13]. In liver cells, both high fructose and high-fat diets elicit hepatic stress responses and activation of pro-inflammatory cascades that lead to insulin resistance. Sucrose-fed rats demonstrate an early alteration of hepatic VLDL-TG secretion, leading to impaired insulin-mediated suppression of glucose production in hepatic tissues after 1–2 weeks, but show no changes in extra-hepatic insulin sensitivity after this time period [33]. After 4–6 weeks, impaired extra-hepatic insulin sensitivity, in conjunction with muscle lipids occurs [33].

The mechanism by which intercellular lipids cause insulin resistance in both liver and muscle is through diacylclycerol (DAG)-induced activation of novel protein kinase C (nPKC) [34]. DAG is a known activator of nPKC, and both DAG and nPKC are associated with lipid-induced insulin resistance in humans [34]. Activation of nPKC causes a decrease in insulin receptors or insulin receptor substrate 1 (IRS1) tyrosine phosphorylation [13]. This IRS-1 inhibition decreases insulin-stimulated glucose transporter (GLUT 4) activity resulting in reduced glucose uptake into the cell [35]. Increases in DAG also activate several other serine/threonine kinases such as inhibitory κβ kinase β (IKKβ) and nuclear factor κB (NF-κB). These inflammatory markers are also activated by tumor necrosis factor-α (TNF-α) and interleukin 6 (IL-6), both known to down-regulate IRS-1 phosphorylation [35]. This is in contrast to a healthy cell in which case insulin binds to its receptor, and causes auto-phosphorylation of the receptors. The phosphorylated receptor then phosphorylates the IRS on the tyrosine residues. The phosphorylated IRS recruits a variety of second messenger proteins, initiating a complex signaling cascade which involves Akt/PKB (protein kinase B) stimulation of glucose uptake into the cell. Insulin sensitivity is thus maintained as a result of enhanced glycogen synthesis, suppression of hepatic gluconeogenesis, increased fatty acid and triglyceride synthesis and suppression of lipolysis in adipose tissue [36]. Cortright et al. [37] found in isolated human skeletal muscles strips and adipocytes that activation of PKC reduced insulin-stimulated glucose uptake; whereas pharmacological inhibition of PKC activity increased insulin-stimulated glucose uptake by 2-fold. This increase was associated with elevated insulin receptor tyrosine phosphorylation of (phosphatidylinositide 3-kinases (PI 3-kinase) activity. Hence, inappropriate activation of PKC may interfere with insulin action by promoting serine/threonine phosphorylation of IRS-1, resulting in prevention of tyrosine phosphorylation of these proteins that is necessary for adequate function on the insulin-signaling pathway [37].

Human research investigating the effects of fructose on insulin sensitivity is limited but the animal literature is more extensive. Specifically, when rats consumed 35% energy as fructose for four weeks, reduced insulin sensitivity associated with impaired hepatic insulin action and whole-body glucose disposal occurred [38]. Although fructose does not increase insulin acutely, the long-term consumption of fructose seems to result in insulin resistance [38,39]. Similarly, rats fed 15% energy from fructose for 15 months displayed elevated fasting serum insulin and glucose concentrations. These results were in conjunction with a more recent animal study in which mice were fed an isocaloric, standard diet; a 60% glucose diet; or a 60% fructose diet for twelve weeks. Glucose disposal was reduced in the fructose fed animals, which resulted in a 1.3-fold lower glucose-stimulated increase in insulin. From these results, and more recent research by Yoo et al [39], a high-fructose diet results in a reduced glucose-stimulated insulin release and impaired glucose disposal [39,40].

In humans, there is limited research confirming the negative effects of fructose on insulin sensitivity and glucose intolerance in adults and adolescents. Sunehag et al. [41] discovered no change in insulin sensitivity or secretion in obese subjects on a high fructose diet, however, the subjects were insulin resistant to start with. In order to maintain substrate homeostasis, normal rates of glucose production, gluconeogenesis, lipolysis and appropriate substrate oxidation, the obese subjects required a more than 2-fold increase in their insulin secretion as compared to what would have been needed had lean adolescents been studied [41]. Similarly, Le et al. [15] found that moderate fructose (1.5 g/kg of body weight) intake for four weeks in seven male subjects induced significant increases in plasma TGs, and VLDL-TG with no change in insulin sensitivity or ectopic fat deposition. The authors speculated that the duration of fructose consumption may need to be longer than 4 weeks in order for the increases in plasma TGs and VLDL-TGs to affect insulin sensitivity [15]. In contrast, Dirlewanger et al. [42] investigated the effects of an acute fructose infusion on hepatic insulin sensitivity during moderate hyperglycemic conditions in ten healthy adults. The infusion with fructose resulted in alterations in endogenous glucose production such that insulin requirements increased 2.3-fold above the two other infusions in order to maintain blood glucose levels [42]. The increased total glucose output indicated that the absolute rate of glucose-6-phosphate hydrolysis and release of free glucose from the liver cells was increased during fructose infusion. Simultaneously, glucose cycling was increased, indicating enhanced reuptake and phosphorylation of glucose by the liver cells. Therefore, an acute fructose infusion induces both extrahepatic and hepatic insulin resistance, with the latter being secondary to an increased intrahepatic glucose 6-phosphate synthesis [42]. Researchers have proposed that the increased hepatic lipid accumulation resulting from fructose-induced DNL would lead to hepatic insulin resistance by increasing levels of DAG. Increases in both DAG and novel PKC are associated with lipid-induced insulin resistance [19,42]. After assessing insulin sensitivity with deuterated glucose disposal prior to and after the 10-week intervention, Stanhope et al. [19] determined that the fructose group had significantly higher fasting insulin and glucose levels as well as increased insulin excursions and endogenous glucose production as compared to the glucose group. Additionally, DNL was significantly higher in the fructose-fed group than the glucose-fed group after the 10-week intervention. These results indicated that hepatic insulin resistance was most likely due to increased DNL from increased DAG and novel PKC [19].

The most commonly proposed mechanism for the fructose-induced insulin resistance appears to be the diminished ability of insulin to suppress hepatic glucose output and decrease insulin receptor density apparent in skeletal muscle and liver [43]. Catena et al. [43] found that insulin receptor number and messenger RNA (mRNA) levels were significantly decreased in skeletal muscle and liver of fructose-fed rats (66% fructose) after two weeks when compared to control rats. These findings suggested that a down-regulation of insulin receptor gene expression is a possible molecular mechanism for insulin resistance. Moreover, abnormalities in insulin action at a post-receptor level in muscles and liver with fructose consumption may also occur, such as decreased phosphorylation of IRS-1 and decreased associated of IRS-1 with PI 3-kinase [44]. This evidence shows that these early steps in insulin signaling are important for insulin’s metabolic effect [43,44]. Therefore, it is concluded that the mechanisms behind fructose-induced insulin resistance are possibly due to the combination of various factors such as a reduction in the number of insulin receptors in skeletal muscle and liver as well as decreased phosphorylation, both caused by increased fat production [43,44,45].

## 6. Fructose and Inflammation

Tumor necrosis factor (TNF)-α, interleukin (IL)-6 and c-reactive protein (CRP), are important pro-inflammatory cytokines induced by elevated triglyceride concentrations which have been linked to insulin resistance [46]. Increases in postprandial TGs and glucose stimulate the activation of neutrophils, leading to an increase in pro-inflammatory cytokines such as IL-6 and TNF-α [47]. IL-6 leads to increased insulin resistance by blocking the IRS-mediated insulin signaling in hepatocytes and muscle cells causing impaired insulin-stimulated glucose uptake into muscle cells [48]. Although the exact mechanism as to how IL-6 affects IRS receptors is not completely understood, it could involve the activation of tyrosine phosphatase or an interaction between suppressor of cytokine signaling (SOCS) proteins and the insulin receptor itself [49]. One of the primary effects of IL-6 is to induce the production of hepatic CRP, which is a known independent risk factor of cardiovascular disease [46]. CRP is an acute phase reactant inflammatory protein which reflects systemic low-grade inflammation [50]. Elevated levels of IL-6 and CRP levels among individuals with features of the insulin resistance and type 2 diabetes have been apparent [51]. Given IL-6’s position in the cytokine cascade as a key mediator of downstream inflammatory processes including activation of coagulation, hepatic release of acute phase reactant proteins, IL-6 may have a potential causal role in metabolic risk factors associated with type 2 diabetes and cardiovascular disease.

Previous studies have shown that increased consumption of fructose results in hyperlipidemia accompanied by insulin resistance and elevated plasma TGs, all leading to increased inflammation [21,52,53]. Specifically, rats fed a diet of 30% fructose for eight weeks experienced increased lipid peroxidation and elevated hepatic TNF-α mRNA expression when compared to all other conditions [53]. Lipid peroxidation led to induction of nitric oxide synthase (NOS) and TNF-α expression in the liver when exposed to high levels of fructose. Moreover, the chronic intake of fructose, and to a lesser extent sucrose, caused significant liver stenosis and increased neutrophil production [53]. Additionally, phosphorylation status of Akt in the liver was altered in mice fed the fructose solution; however, a similar effect of fructose feeding was not found in the TNF-α knockout mice. This implies that TNF-α may be critical in mediating insulin resistance in mice chronically fed fructose [53]. It has been suggested that an induction of TNF-α may suppress the activation of AMP-activated protein kinase (AMPK) in the liver [54]. Kanuri et al. [52] found similar results when wild-type mice or TNF-α knockout mice were fed a 30% fructose solution or tap water for eight weeks. The fructose-fed, wild-type mice had significantly higher TG accumulation, which resulted in a 5-fold increase from baseline values. Moreover, the fructose-fed mice had significantly higher neutrophil infiltration; whereas in the fructose-fed TNF-α knockout mice, the neutrophil infiltration was similar to in the water-fed controls [52]. In the fructose-fed TNF-α knockout mice, hepatic stenosis and neutrophil infiltration was attenuated, which resulted in increased phosphorylation of AMPK and Akt, similar to the water-fed controls. Since phosphorylation status of Akt in the liver was altered in the fructose-fed mice wild-type mice and not the TNF-α knockout mice, it was concluded that TNF-α and its receptor 1 may be critical in mediating insulin resistance in the mice chronically fed fructose [52,55]. In a longer duration study, Sanchez-Lozada et al. [56] investigated whether a drink containing 30% glucose with 30% fructose or 60% sucrose induced fatty liver when compared to rats fed a standard chow diet for 16 weeks [56]. Liver inflammation was induced as a result of elevated TNF-α with both the fructose + glucose diet as well as the sucrose diet when compared to the control group (standard chow). The increases in inflammatory markers significantly correlated with increases TG levels as well [56].

The aforementioned studies [52,53,54,55,56] have indicated that increases in inflammatory markers such as TNF-α can create changes in insulin signaling which can be exacerbated with fructose ingestion. Although there is a lack of direct experimental evidence linking fructose and inflammation, the process of lipid accumulation within the liver may induce a sub-acute inflammatory response that is similar to that seen in obesity-related inflammation within adipocytes. TNF-α, IL-6 and IL-1β, all pro-inflammatory markers, are overproduced in fatty liver and participate in the development of insulin resistance and activate hepatic macrophages called Kupffer cells [57]. Unlike adipose tissue in which macrophages are relatively sparse in a basal state and increase with increased adiposity, the liver is densely populated with Kupffer cells. Toll-like receptor 4 (TLR4) and cluster of differentiation 14 ( CD14, receptors on the Kupffer cell that internalize endotoxins activate the transcription of pro-inflammatory cytokines such as TNFα and interleukins [57]. More research needs to be conducted to fully elucidate the impact that fructose has on inflammation.

## 7. Physical Inactivity and Fructose Consumption

### 7.1. Physical Inactivity

Physical inactivity and poor cardiovascular fitness has been consistently associated with an increased risk of chronic diseases such as type 2 diabetes and cardiovascular disease [58]. Being physically inactive and/or unfit is associated with many health consequences and is an important component of a comprehensive approach to disease prevention and health promotion [59]. Observational studies have demonstrated that the most unfit individuals are at the greatest risk of chronic diseases and all-cause mortality regardless of their gender, race, ethnic background or weight [60]. Therefore, preventing metabolic risk factors such as coronary artery disease, type 2 diabetes and hyperlipidemia can be accomplished by incorporating moderate activity into a person’s daily routine in order to avoid the ill effects of physical inactivity [61,62].

In 1953, Morris et al. [63] determined that workers who were seated most of the day, such as bus drivers and telephonists, were twice as likely to develop cardiovascular disease than workers who stand or are ambulatory most of the work day such as mail carriers. This study was reproduced more recently in 2005 [64] in an epidemiologic study of 73,743 postmenopausal women from the Woman’s Health Initiative Study in which those who were inactive had increased risk of cardiovascular disease, and this was reversed with increased physical activity [64]. The Australian Diabetes, Obesity and Lifestyle Study also reported that sitting time and self-reported television viewing was positively correlated with undiagnosed abnormal glucose metabolism [65]. These results persisted after adjustment for sustained and moderate-intensity leisure-time physical activity. A subsequent study from the same Australian cohort found that individuals who reported having participated in the required dose of weekly physical activity (30 min/day, 5 times/week) still had detrimental waist circumference, systolic blood pressure, and 2-hour plasma glucose after correcting for such variables with television viewing time [66]. Clearly, this indicates that focusing on acquiring the recommended dose of exercise is not a strong enough of a stimulant to completely protect the body from physical inactivity the other 23+ h/day. In this same cohort, 1958 adults over the age of 60 years who reported high levels of sedentary behavior, had a greater prevalence of developing metabolic syndrome [67]. This data provides evidence that reducing prolonged overall sitting time may reduce metabolic disturbances. Hence, there is a need for more specific sedentary behavior recommendations and health guidelines for adults in addition to the current recommendations on physical activity [66]. Data from the Medical Expenditure Panel Survey indicated that both physical inactivity and obesity are strongly and independently correlated with diabetes and cardiovascular disease [68]. According to the survey, the likelihood of having diabetes increases with physical inactivity regardless of body mass index (BMI), indicating that it is better to be active than inactive. Hence, both physical inactivity and obesity seem to be independently associated with diabetes and diabetes-related risk factors [68]. Moreover, Healy et al. [61] discovered that adults participating in minimal physical activity had higher glucose concentrations compared to more active individuals, reiterating the findings that physical inactivity alters glucose homeostasis [61].

Not only does prolonged inactivity decrease the opportunity for cumulative energy expenditure resulting from numerous muscle contractions [60], physical inactivity also induces molecular changes. Within six to eight hours of physical inactivity, the suppression of skeletal muscle LPL activity and reduced muscle glucose uptake occur, resulting in elevated plasma TG and reduced high density lipoprotein (HDL)levels [60]. Lipoprotein lipase is an important enzyme involved in the molecular alterations, affecting physical inactivity [69]. LPL is the main enzyme responsible for the breakdown of VLDL-TGs and chylomicrons on the endothelial. LPL also enhances the removal of VLDL by the VLDL receptor and indirectly plays a role in maintaining high levels of plasma HDL cholesterol. Hence, low LPL is associated with blunted plasma TG uptake as well as reduced HDL levels [60]. Local regulation of LPL provides a means of generating a concentrated source of fatty acids as well as other lipoprotein-derived lipids [69]. Moreover, LPL is involved in the regulation of gene expression of inflammatory markers which lead to cardiovascular disease [69]. Because physical inactivity regulates LPL activity in the vasculature and skeletal muscle, reduced physical activity can decrease LPL activity 10–20 fold [62]. However, such decreases can be reversed within several hours of ambulatory contractions, implying that a reduction in contractile activity is a potent physiological factor determining LPL activity [69]. Lipoprotein lipase response to physical inactivity and plasma lipid in the microvasculature of skeletal muscle can best be described by first understanding the mechanisms behind the LPL response during ambulation [70]. During ambulation, the vascular endothelial cells are at the interface with plasma TG and fatty acids bound to albumin. During standing or ambulation, there is high LPL activity in the microvasculature of the skeletal muscle. Physically active muscles have greater rates of TG-derived fatty acid uptake, albumin-bound fatty acid transport, fatty acid oxidation, and intracellular TG synthesis. Moreover, there are reduced concentrations of intramuscular fatty acids and fatty acetyl-CoA [70]. In contrast, during inactivity when normal metabolic processes have slowed down, there is a removal of the local energy demands of physical activity, leading to an elevation in TGs and fatty acids. Plasma fatty acids and TGs accumulate as a result of a lower rate of LPL-induced fatty acid oxidation.

Regulation of LPL activity may be different during states of inactivity versus activity [62]. Normally, high activity of LPL in oxidative muscle significantly decreases with physical inactivity and increased physical activity restores such effects. In conjunction with the changes seen with LPL, the uptake of TGs and high density lipoprotein cholesterol decreases with physical inactivity. Therefore, the steps involved in muscle LPL regulation, which are sensitive to inactivity, can be prevented and even reversed with minimal, non-fatiguing contractions (ex: slow treadmill walking) [62]. Zderic and Hamilton [69] found that inactivity causes a 47% decrease in LPL activity within eight hours and an additional 13% after 12 h of inactivity. Moreover, plasma TG concentrations increase after twelve hours, resulting in significant decreases in LPL activity [69]. They too concluded that decreased physical activity depresses LPL activity and that increased fat intake with ambulation suppresses LPL activity, similar to that of inactivity. These results parallel the previously stated concept that there is an inverse relationship between TG concentrations and LPL activity and that decreased activity amplifies the response.

In the Studies of Targeted Risk Reduction Intervention through Defined Exercise (STRIDDE), the researchers investigated whether the training-induced benefits in serum lipids and lipoproteins are sustained over five and/or fifteen days of exercise detraining [71]. Subjects were randomized into one of four groups: (1) high amount/vigorous intensity (caloric equivalent to approximately 20 minutes per week at 65–80% peak oxygen consumption; (2) low amount/vigorous intensity equivalent to approximately 12 m per week at 65% to 80% peak oxygen consumption; and (3) low amount/moderate intensity with a caloric equivalent of approximately 12 m per week at 40–55% peak oxygen consumption and (4) a control non-exercising group for six months. The modest-intensity training group reduced total TGs and VLDL-TG at 24-h post-exercise training by twice the magnitude of the two more vigorous exercise-training groups. In the two vigorous-intensity training groups, total TGs and VLDL-TGs had returned to baseline after only 5 days, indicating that there was no sustained TG-lowering effect in those two groups. While the mechanisms for the aforementioned effects were unclear, the authors speculated that exercise of different intensities may have tissue-specific effects on the LPL bound to the endothelial cells, resulting in differential effects of exercise of varying intensities on TG, VLDL and HDL metabolism [71]. Subsequently, endurance athletes are also not protected from physical in activity. Herd et al. [72] put endurance-trained subjects on a detraining program and discovered that within 60 h after the last training session, the runners’ lipidemic response to a fat load was 37% higher than baseline, and 46% higher after 9 days of detraining. These changes correlated with a reciprocal decrease in LPL activity [72]. This data supported the previous hypothesis that hydrolysis at the endothelial surface of capillaries by LPL is the rate-limiting step in TG clearance, and changes in LPL activity with changes in exercise or training status are most likely the cause of the above findings [72].

As seen in the previous research, physical inactivity creates a significant deleterious metabolic state in which insulin sensitivity is decreased within a few hours of detraining. Physical activity improves insulin sensitivity both acutely and chronically as a result of changes in insulin signaling. This process is not mediated by the insulin-dependent rapid phosphorylation of the insulin receptors [69,73,74]. In contrast, exercise stimulates an insulin-independent pathway. With muscle contraction, glucose uptake is mediated by multiple signaling pathways such as protein kinase-C, Ca^+2^/calmodulin-dependent protein kinase (CaMKK) and AMPK [69]. The translocation of glucose receptors (GLUT 4) to the cell membrane occur because of increased Akt activity and phosphorylation within the cell [75]. This effect is short-lived, lasting 48–72 h; therefore, to maximize the benefits of physical activity on insulin sensitivity exercise should be repeated within this timeframe.

### 7.2. Physical Activity and Inflammation

Although exercise causes an acute inflammatory response [76], physical activity and improved cardiovascular fitness decreases low-grade inflammation by decreasing body fat, decreasing chronic production of pro-inflammatory cytokines and increasing production of anti-inflammatory cytokines. Moreover, exercise reduces expression of adhesion molecules, up-regulates antioxidant and other cellular defenses and improves endothelial function [77]. Although low-grade inflammation, characteristic of elevated IL-6 levels, has been associated with obesity and insulin resistance, it is markedly produced and released after an acute bout of exercise. However, IL-6 may actually help to prevent or reduce risk factors associated with metabolic syndrome and type 2 diabetes in the long-term [75]. During exercise, the magnitude of the increase in IL-6 is relative to the duration, intensity of exercise and amount of muscle mass involved. Muscle biopsies from humans and rats have demonstrated increases in IL-6 after exercise up to 100 times that of resting values [78]. In response to muscle contraction, both type I and type II muscle fibers express IL-6, which exerts its effects both locally and peripherally in several organs of the body when released into circulation. IL-6 may also work in an endocrine manner to increase hepatic glucose production during exercise or during lipolysis in adipose tissue [79]. The anti-inflammatory effects of IL-6 have been demonstrated by the ability of IL-6 to stimulate the release of classical anti-inflammatory cytokines such as IL-1ra (receptor antagonist) and IL-10 [78]. Hence, IL-6 has both pro- and anti-inflammatory properties. When IL-6 is signaling monocytes or macrophages, the activation of nuclear factor (NF-κB) and TNF-α occurs, leading to an inflammatory state but when IL-6 is released from muscle, it creates an anti-inflammatory state [75]. Therefore, the possibility exists that the long-term effect of exercise may be a result of the anti-inflammatory process of an acute bout of exercise. For that reason, acute exercise will protect against chronic systemic low-grade inflammation, and thereby offer protection against insulin resistance and atherosclerosis.

### 7.3. Fructose Ingestion and Physical Activity

For athletes, fructose provides a beneficial aid in training due to its ability to stimulate rapid nutrient absorption in the small intestine and help increase exogenous carbohydrate oxidation during exercise [6]. When fructose is mixed with glucose in sports drinks, carbohydrate oxidation is enhanced by 40%. This dramatic increase in oxidation can be explained by the different transport systems used for intestinal absorption. Moreover, fructose has been shown to reduce the perception of fatigue and stress during exercise, and improve exercise performance during cycling exercises [80].

Although fructose consumption may pose an advantage in an athletic environment, it does not seem to be warranted for the general public. To date, there are only two reports of fructose and physical activity. Botezelli et al. [27] studied 48 Wister rats to determine whether aerobic exercise alters markers of fatty liver disease when fed a diet high in fructose. Thirty days of aerobic exercise resulted in the fructose-fed rats having altered metabolic profiles which included elevated plasma TG as a result of the fructose diet. However, they had improved insulin sensitivity and decreased cholesterol levels, resulting from the exercise regimen. The changes in TG levels were most likely due to the improved lipid oxidation and availability of circulation TG as a result of exercise [27]. In a more recent study in young, healthy individuals, consumption of an additional 75 g of fructose per day in conjunction with physical inactivity (~4200 steps/day) resulted in increased postprandial lipidemia and precursors to low-grade inflammation, whereas when physical activity was increased to ~12,000 steps/day, these effects were ameliorated [81]. Hence, increased physical activity may improve features of fructose-induced metabolic syndrome. More studies on humans still need to be conducted to determine the interaction between fructose consumption and physical activity, however, preliminary research indicates that increasing ones’ physical activity may counteract the adverse effects of a fructose-rich diet.

## 8. Conclusions

Although it seems apparent that increased intake of fructose leads to various risk factors associated with metabolic syndrome such as hypertension, hyperlipidemia, insulin resistance, inflammation and hyperuricemia, there is still numerous contradictory evidence which states that as long as fructose is consumed in moderate doses, fructose may not augment these risk factors. The quantities of fructose administered in many of the studies used concentrations that were well above the average fructose intake of 60–70 g/day, and with increased daily caloric intake, which may have differing results. Hence, there is a need for future research investigating the effects of fructose when using quantities that more closely match that of the average population. Moreover, there is very limited research indicating how physical inactivity may confound these risks. Although fructose consumption cannot be completely to blame for the increased rates of obesity and metabolic syndrome, fructose is often associated with additional detrimental behaviors such as a hypercaloric diet, or a diet rich in saturated fats, as well as low physical activity [6]. These behaviors lead to risk factors of metabolic syndrome, and as such could be prevented and/or reduced.

The above review of literature summarizes the proposed mechanisms associated with the fructose-induced metabolic alterations related to metabolic syndrome. These risk factors, such as postprandial hyperlipidemia, insulin resistance, and hyperuricemia, seem to be exacerbated with fructose ingestion in a dose-dependent manner; hence continued research must be conducted to completely elucidate the importance of decreasing fructose consumption. Specifically, this includes research into whether ingesting large amounts of fructose, with an ab libitum diet, will cause changes in circulation LPL concentrations. Furthermore, compounding the increased fructose consumption with a sedentary lifestyle may be exacerbating the fructose-induced metabolic disturbances, therefore, more research should be conducted to determine whether increasing physical activity will improve LPL activity in a fructose-fed individual. Although there is still too little data to suggest, at this point, that increased physical activity can attenuate these metabolic disturbances, increasing one’s physical activity, regardless of the amount of structured exercise that is performed, should be a national priority, as the minimal amount of research regarding fructose and physical activity is positive.
